# Supplementary material for: A high-resolution mRNA expression time course of embryonic development in zebrafish
Source: eLife. 2017 Nov 16;6:e30860. doi: 10.7554/eLife.30860 (PMC5690287; doi:10.7554/eLife.30860)
Supplement: Supplementary file 6. [file elife-30860-supp6.zip › biolayout-clusters-files/Cluster236-genes.html]

Cluster236


# Cluster236: Genes

| Ensembl ID | Gene Name | Chr | Start | End | Biotype |
| --- | --- | --- | --- | --- | --- |
| ENSDARG00000043035 | capn3b | 20 | 26056028 | 26102200 | protein\_coding |
| ENSDARG00000097959 | si:dkey-248g15.3 | 17 | 2373117 | 2378829 | protein\_coding |
| ENSDARG00000096667 | si:dkey-25o16.2 | 1 | 25974158 | 25977496 | protein\_coding |
| ENSDARG00000101393 | si:dkey-31g6.6 | 6 | 21892727 | 21895018 | protein\_coding |
| ENSDARG00000105341 | si:dkey-9l20.3 | 23 | 3595560 | 3698536 | protein\_coding |
| ENSDARG00000087318 | zgc:174688 | 7 | 8133875 | 8135474 | transcribed\_unprocessed\_pseudogene |
